# Effects of exercise on brain activity during walking in older adults: a randomized controlled trial

**DOI:** 10.1186/s12984-017-0263-9

**Published:** 2017-05-30

**Authors:** Hiroyuki Shimada, Kenji Ishii, Hyuma Makizako, Kiichi Ishiwata, Keiichi Oda, Megumi Suzukawa

**Affiliations:** 1 Department of Preventive Gerontology, Center for Gerontology and Social Science, National Center for Geriatrics and Gerontology, 7-430 Morioka-cho, Obu, Aichi 474-0038 Japan; 2 Research Team for Neuroimaging, Tokyo Metropolitan Institute of Gerontology, 35-2 Sakae-cho, Itabashi-ku, Tokyo, 173-0015 Japan; 3 Department of Physical Therapy, University of Human Sciences, 1288 Magome, Iwatsuki-ku, Saitama, 339-8539 Japan; 4 Department of Physical Therapy, School of Health Sciences, Faculty of Medicine, Kagoshima University, 8-35-1 Sakuragaoka, Kagoshima, 890-8544 Japan; 5 Department of Radiological Technology, Faculty of Health Sciences, Hokkaido University of Science, Sapporo, Japan

**Keywords:** Regional brain activation, Brain function, Elderly, FDG-pet, Walking

## Abstract

**Background:**

Physical activity may preserve neuronal plasticity, increase synapse formation, and cause the release of hormonal factors that promote neurogenesis and neuronal function. Previous studies have reported enhanced neurocognitive function following exercise training. However, the specific cortical regions activated during exercise training remain largely undefined. In this study, we quantitatively and objectively evaluated the effects of exercise on brain activity during walking in healthy older adults.

**Methods:**

A total of 24 elderly women (75–83 years old) were randomly allocated to either an intervention group or a control group. Those in the intervention group attended 3 months of biweekly 90-min sessions focused on aerobic exercise, strength training, and physical therapy. We monitored changes in regional cerebral glucose metabolism during walking in both groups using positron emission tomography (PET) and [^18^F]fluorodeoxyglucose (FDG).

**Results:**

All subjects completed the 3-month experiment and the adherence to the exercise program was 100%. Compared with the control group, the intervention group showed a significantly greater step length in the right foot after 3 months of physical activity. The FDG-PET assessment revealed a significant post-intervention increase in regional glucose metabolism in the left posterior entorhinal cortex, left superior temporal gyrus, and right superior temporopolar area in the intervention group. Interestingly, the control group showed a relative increase in regional glucose metabolism in the left premotor and supplemental motor areas, left and right somatosensory association cortex, and right primary visual cortex after the 3-month period. We found no significant differences in FDG uptake between the intervention and control groups before vs. after the intervention.

**Conclusion:**

Exercise training increased activity in specific brain regions, such as the precuneus and entorhinal cortices, which play an important role in episodic and spatial memory. Further investigation is required to confirm whether alterations in glucose metabolism within these regions during walking directly promote physical and cognitive performance.

**Trial registration:**

UMIN-CTR (UMIN000021829). Retrospectively registered 10 April 2016.

## Background

Numerous studies have demonstrated the beneficial effects of exercise in older adults [1], including those with chronic disorders leading to functional decline [2]. Several meta-analyses and randomized controlled trials have reported that physical activity is associated with improvements in attention, processing speed, and executive function [3, 4] as well as sensorimotor ability in older adults. Indeed, aerobic exercise may lead to an increase in brain volume [5, 6] and enhance functional connectivity between parts of the frontal, posterior, and temporal cortices [7] in healthy older adults. For example, Erickson et al. found that the hippocampus remains plastic in late adulthood and that a 1-year period of aerobic exercise was sufficient to increase hippocampus volume [5]. Although the physiological mechanisms underlying exercise-induced improvements in physical performance are well understood, the relationship between exercised-induced improvements in physical performance and changes in brain activity remains unclear.

In comparison to low-fitness or nonaerobic control participants, functional magnetic resonance imaging (fMRI) studies have shown that fit or aerobically trained older adults have greater functional connectivity between parts of the frontal, posterior, and temporal cortices [7]. This enhanced functional connectivity extends to task-related activities in regions of the prefrontal and parietal cortices involved in spatial selection and inhibitory function [8]. Thus, exercise appears to improve regional brain activity and assist in maintaining cognitive function in older adults. A number of imaging studies have measured glucose metabolism in the brain during walking using positron emission tomography (PET) with [^18^F]fluorodeoxyglucose (FDG) [9, 10]. Some studies have used single-photon emission tomography with technetium-99 m hexamethyl propylene amine oxime or ^99m^Tc-ethyl cysteinate dimer to measure the fixed regional cerebral blood flow [11, 12], while other studies have used near-infrared spectroscopy to reflect blood oxygenation changes following neuronal activity [13]. All of the above-cited studies reported activation of the medial frontoparietal region, supplementary motor area, lateral premotor cortex, cingulate cortex, superior parietal lobule, precuneus, and infratentorial region [9–12] during walking. However, no studies have investigated whether this activation occurs as a result of exercise training.

Consequently, in this study, we sought to clarify the effects of an exercise intervention program on gait function and brain activity during walking in older adults. We hypothesized that an exercise regimen including aerobic, resistance, and balance exercises may be effective in improving physical function and enhancing brain activity. We used a randomized control trial design with FDG-PET to measure brain activity before and after an exercise intervention. This study was recruited the healthy older women, because it is evident that there are sex influences at all levels of the nervous system, from genetic to systems to behavioral levels [14].

## Methods

### Participants

**Fig. 1:**
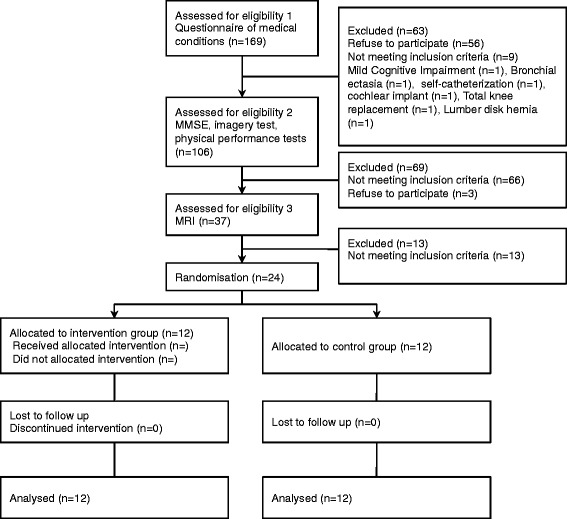
Flow diagram of the experimental procedure. MMSE, Mini Mental State Examination; MRI, magnetic resonance imaging; PET, positron emission tomography

We selected 274 potential female subjects who were ≥75 years old, lived in Tokyo, and had no history of neurological or psychiatric disorders, cardiovascular disease, hypertension, heart failure, diabetes mellitus, head trauma, drug or alcohol abuse, or severe pain from a database of elderly volunteers (*n* = 1289), generated in April 2009. Of 106 elderly women who completed cognitive and physical performance tests, 69 were excluded due to low cognitive function (i.e., Mini Mental State Examination score < 27) [15], use of multiple medications, a drug allergy, or gait disturbance. Of the remaining 37 women, 13 were excluded due to abnormal signal intensities or evidence of brain atrophy, as revealed by magnetic resonance imaging (MRI) with T1-weighted contrast using a 1.5-T Signa Horizon scanner (GE, Milwaukee, WI). A radiologist determined these abnormalities based on visual inspection. Thus, the remaining 24 healthy elderly women were chosen to participate in this study (mean age, 78.0 ± 2.3 years; range, 75; mean height, 147.7 ± 3.8 cm; mean weight, 49.7 ± 4.9 kg) (Fig. 1).

### Experimental design

Prior to commencing the study, we conducted assessments of the participants to establish a baseline. Researchers who were blinded to the aims of the study then performed a randomization in which participants were randomly assigned to either an intervention or control group in a 1:1 ratio using the “random sample of cases” option in IBM SPSS Statistics software (Version 19; SPSS Inc., Chicago, IL). The researchers involved in data collection were also blinded to the group assignment for each subject. PET scan and physical examinations such as gait measurements were performed different days. The participant characteristics are summarized in Table 1.

**Table 1:** Comparison between anthropometric and gait measures at baseline in the intervention and control groups

	Intervention group	Control group	*p* value
Age, years	78.1 ± 2.4	78.0 ± 2.3	.93
Height, cm	145.6 ± 3.3	149.9 ± 3.2	< .01
Weight, kg	51.3 ± 4.9	48.2 ± 4.5	.13
SBP, mmHg	142.2 ± 20.0	134.7 ± 18.4	.35
DBP, mmHg	81.2 ± 6.5	82.0 ± 11.0	.82
Grip strength, kg	21.6 ± 5.3	22.1 ± 3.1	.78
Stance phase time, s	1.2 ± 0.1	1.2 ± 0.1	.98
Swing phase time, s	0.7 ± 0.1	0.6 ± 0.1	.57
Double stance phase time, s	0.2 ± 0.02	0.2 ± 0.02	.12
CWS, cm/s	125.6 ± 14.5	134.9 ± 14.0	.12
Step length of left foot, cm	58.3 ± 6.2	63.6 ± 5.5	.04
Step length of right foot, cm	58.2 ± 6.0	63.7 ± 5.7	.03
Cadence, steps/min	129.8 ± 12.0	128.9 ± 13.2	.85
MTA scale, n (%)			
Score 0 (normal)	9 (75.0)	9 (75.0)	1.00
Score 1	3 (25.0)	3 (25.0)	
PVH, n (%)			
Grade 0 (absence)	9 (75.0)	7 (58.3)	.54
Grade 1	2 (16.7)	2 (16.7)	
Grade 2	1 (8.3)	3 (25.0)	
DSWMH, n (%)			
Grade 0 (absence)	6 (50.0)	6 (50.0)	1.00
Grade 1	5 (41.7)	5 (41.7)	
Grade 2	1 (8.3)	1 (8.3)	

*SBP* Systolic blood pressure, *DBP* Diastolic blood pressure, *CWS* Comfortable walking speed, *MTA* Medial temporal lobe atrophy, *PVH* Periventricular hyperintensity, *DSWMH* Deep and subcortical white matter hyperintensity

We used FDG-PET to assess brain glucose metabolism during walking before and after the intervention. All subjects were asked to refrain from consuming sugar for at least 6 h and to avoid strenuous physical activity for at least 2 days before the FDG-PET examination. The examination contained three phases: preparation (40 min), exercise (25 min), and a PET scan. During the preparation phase, a catheter was inserted into a forearm vein for the purpose of injecting FDG or drawing blood. Venous blood samples were obtained during the preparation period, at the end of the preparation period, and 10 min after the end of the walk to assess blood glucose concentration. During the exercise phase, subjects walked for 25 min at 2.0 km/h on a treadmill (PW-21; Hitachi, Tokyo, Japan). They were asked to hold the handrails to avoid falling and to ensure a uniform visual environment. Subjects then rested on a bed with their eyes closed for 10 min. After the exercise phase, PET scans were performed using a Headtome-V (SET 2400 W; Shimadzu, Kyoto, Japan) in the three-dimensional mode.

### Physical exercise intervention

Subjects in the intervention group participated in a 3-month physiotherapy program conducted by two physiotherapists trained in geriatric rehabilitation. This program involved a total of 40 biweekly, 90-min sessions focused on aerobic exercise, muscle strength training, and postural balance retraining. Each supervised session involved six participants and began with 10 min of warm-up and stretching exercises followed by 20 min of strength training exercises. For the final 60 min, the subjects engaged in circuit training in which they completed individually prescribed muscle exercises, gait retraining, stair climbing and descending, postural control training, realignment of standing posture, and aerobic exercise using a bicycle ergometer. Physiotherapists conducted risk assessments for subjects before and after each session.

The physiotherapists and a well-trained instructor implemented a risk management component of the physiotherapy program. The subjects were instructed to carry out daily home-based muscle strength and walking exercises, all of which were self-monitored using a booklet and pedometer. Attendance at each session was recorded and a transportation service was provided for participants, where necessary, to ensure compliance. The control group was not received the intervention.

### Outcomes

#### Gait performance

Gait variables were measured using a WalkWay device (WalkWay MW-1000; Anima, Tokyo, Japan), which measures the distribution of foot pressure during walking [16]. The WalkWay measures pressure applied to a surface of 800 × 2400 mm (5 mm thick) and is mounted with strain gauges placed 10 mm apart (14,000 points). To ensure that the WalkWay device measured gait according to the normal walking pace of each subject, participants were required to walk for at least 1.5 m before and after approaching the device. This process was repeated for a total of 5 times to ensure consistency. Three temporal gait parameters and four spatiotemporal gait parameters were calculated from the distribution of foot pressure. Stance phase time, swing phase time, and double stance phase time were calculated as temporal parameters, while gait speed, step length for each side, and cadence were calculated as spatiotemporal measures.

#### Image acquisition and processing

At the onset of walking, FDG (180 MBq) was injected intravenously through the catheter. A 6-min emission scan was obtained 40 min after the injection to create images with the following parameters: matrix size, 96 × 96 × 50 mm; voxel size, 2 × 2 × 3.125 mm. The images were reconstructed using a filtered backprojection algorithm with a second-order low-pass filter and cutoff frequency of 1.25 cycles/cm. Corrections were applied for dead time and detector non-uniformity. Image processing and data analyses were performed using statistical parametric mapping (SPM) (SPM8 software; Welcome Department of Cognitive Neurology, Institute of Neurology, Queen Square, London, UK) in MATLAB (MathWorks, Natick, MA). The tasks performed using SPM8 included MRI/PET coregistration, spatial normalization, spatial smoothing, MRI segmentation, and SPM analysis. Anatomical brain MR images were spatially normalized to the Montreal Neurological Institute (MNI, McGill University, Montreal, Canada) standard template using an affine transformation (12 parameters for rigid transformations) [17]. These parameters were then applied to the coregistered FDG-PET images. All stereotactic coordinates used in this study refer to the MNI coordinate system. The spatially normalized images were blurred using a Gaussian filter (full width at half maximum of 12 mm) to increase the signal-to-noise ratio. All scans were analyzed after normalization for white matter. Specifically, prior to voxel-based statistical analysis, normalization was conducted using an anatomical mask in MNI space, which served to remove the effect of differences in the overall counts [18]. To stabilize the variance related to the substantial differences in global activity between high- and low-dose images, we normalized the pixel values by scaling the activity in each pixel in proportion to the global activity. In this process, the mean global activity of each scan was adjusted to 50 [12]. We then performed planned comparisons before and after the intervention using *t* statistics for each voxel. These analyses generated statistical parametric maps of the *t* statistic (SPM[*t*]), which were subsequently converted to the unit normal distribution (SPM[Z]). The estimated final spatial resolution was 19 × 21 × 18 mm. We assessed the presence of medial temporal lobe atrophy (MTA) and white matter hyperintensity (WMH) at baseline, as revealed by MRI with T1- and T2-weighted contrast. The five-point MTA scale [19] and Fazekas scale [20] were used to determine the MTA atrophy, and periventricular hyperintensity (PVH) and deep and subcortical white matter hyperintensity (DSWMH), respectively. Two neurologists determined these abnormalities based on visual inspection.

#### Analysis

We performed statistical analysis for gait performance using IBM SPSS statistics software. We estimated that with 24 participants, the power of the study would be such that there would be a 90% chance of detecting a significant between-group difference in the change in gait speed, with a moderate effect size of 0.35. For baseline comparisons, we compared the basic characteristics and MTA atrophy and WMH of patients between the two groups using *t*-tests or Chi square test. Gait performance before and after the 3-month intervention period for each group was also compared using *t*-tests. A repeated-measures analysis of covariance (ANCOVA) was used to determine between-group differences. The interactions between groups were also examined in the ANCOVA analyses, which included age and height as covariates. All tests for statistical significance were two-sided, and an alpha-level of 0.05 was considered statistically significant.

In the FDG-PET analysis, the activated brain regions were identified according to stereotaxic coordinates and visual inspection of the structural MRI provided by SPM8. To analyze the effects of the exercise intervention, we compared cerebral FDG uptake during walking before vs. after the 3-month intervention program. We also compared cerebral FDG uptake during walking between the intervention and control groups to identify group differences before and after the intervention. A relative increase in glucose metabolism was calculated and considered significant at *p* < 0.05 using a family-wise error (FWE) correction.

## Results

### Study overview

Figure 1 contains a flowchart summarizing the experimental procedure from the time of screening to study completion at 3 months. All subjects completed the 3-month follow-up. One subject from the control group refused the FDG-PET measurement at completion. The mean adherence to the exercise program was 100%. There were no significant differences between the intervention and control groups with respect to baseline characteristics and MTA atrophy and WMH, except for height and step length (Table 1).

### Outcomes for gait performance

**Fig. 2:**
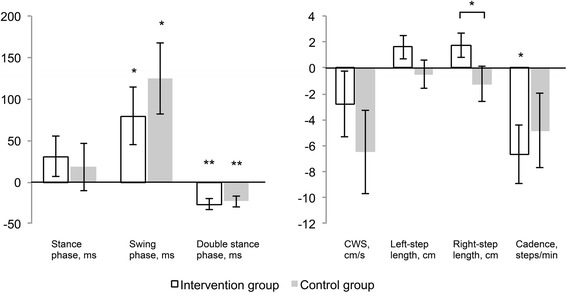
Changes in temporal and spatiotemporal gait performance after the intervention. * *p* < .05, ** *p* < .01; CWS, comfortable walking speed. We observed an increase in swing phase time and a decrease in double stance phase time and cadence in the intervention group. We found an increase in swing phase time and a decrease in double stance phase time, but no change in cadence in the control group. After the intervention, the intervention group showed a significantly greater step length for the right foot

When we compared gait before vs. after the intervention, we found that the swing phase time increased while the double stance phase time decreased after the 3-month period in both groups. Compared with baseline values, the intervention group showed a significant decrease in cadence after the exercise program. Additionally, the intervention group showed a significantly greater step length in the right foot after the program compared with the control group (Fig. 2).

### Outcomes for FDG-PET

**Fig. 3:**
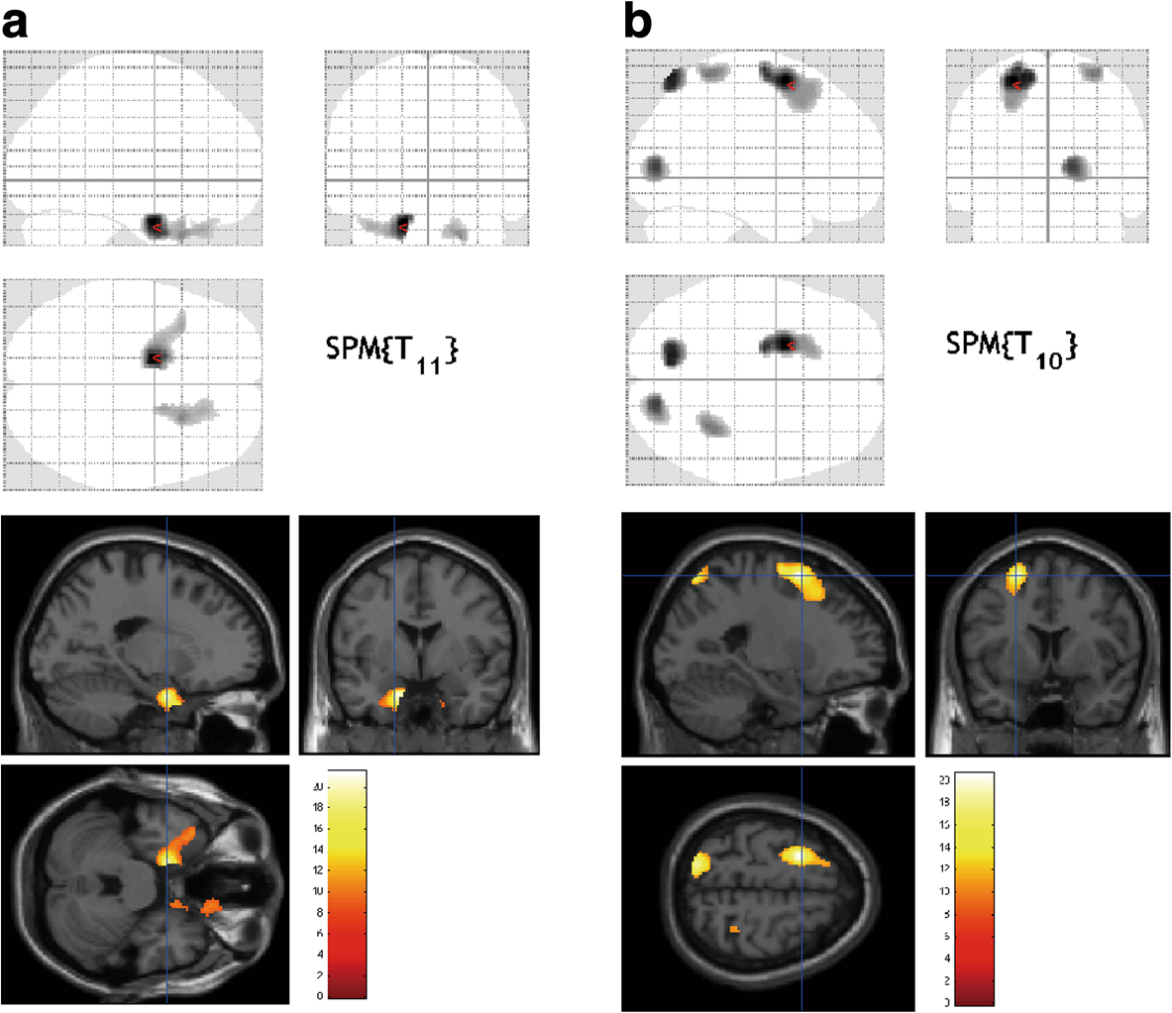
FDG–PET activation during walking (compared with baseline, *p* < 0.05; FWE corrected) in the intervention (**a**) and control (**b**) groups. During walking, activations after the 3-month exercise program were prominent in the left posterior entorhinal cortex (BA 28), left superior temporal gyrus (BA 38), and right superior temporal gyrus (BA 38 and 32) compared with baseline in the intervention group (**a**). In the control group, we observed prominent activations during walking after the 3-month experimental period in the left superior frontal gyrus (BA 6), left superior parietal lobule (BA 7), right cuneus (BA 17), and right postcentral gyrus (BA 5) compared with baseline

In the intervention group, we observed a significant increase in regional FDG uptake in the left posterior entorhinal cortex (*p* = 0.001, FWE-corrected), left superior temporal gyrus (*p* < 0.05, FWE-corrected), and right superior temporopolar area (*p* < 0.01, FWE-corrected) after the 3-month period (Table 2, Fig. 3).

**Table 2:** Scores for different activation areas during walking after the 3-month experimental period compared with baseline measurements

(a) FDG activation during walking in the intervention group (vs. baseline measurement)								
Cerebral hemispheres	BA	Cluster	Z	T	p	x	y	z
Left posterior entorhinal cortex	28	499	6.33	21.47	<0.001	−16	0	−26
Left superior temporal gyrus	38		5.07	10.63	0.011	−38	18	−30
Right superior temporal gyrus, temporopolar area	38	285	5.37	12.45	0.004	22	18	−36
	38		5.16	11.15	0.008	16	4	−32
(b) FDG activation during walking in the control group (vs. baseline measurement)								
Cerebral hemispheres	BA	Cluster	Z	T	p	x	y	z
Left superior frontal gyrus	6	875	6.04	20.62	< 0.001	−24	6	62
			5.74	17.12	0.001	−20	−8	70
			5.24	12.78	0.011	−26	14	50
Left superior parietal lobule	7	259	5.86	18.46	0.001	−16	−70	66
Right occipital lobe, Cuneus	17	360	5.67	16.46	0.002	16	−82	8
Right postcentral gyrus	5	222	5.41	14.07	0.006	30	−46	66

In the control group, we observed a relative increase in regional glucose metabolism in the left premotor and supplemental motor areas (*p* < 0.001, FWE-corrected), left and right somatosensory association cortex (*p* < 0.01, FWE-corrected), and right primary visual cortex (*p* < 0.01, FWE-corrected) after the 3-month period (Table 2, Fig. 3).

When comparing the intervention and control groups, we found no significant increase in regional FDG uptake before and after the intervention. However, during walking after the 3-month period, each group exhibited an increase in regional glucose metabolism in different brain areas. There were no adverse events reported during the study period.

## Discussion

Compared with the baseline assessment, individuals in the intervention group showed a decrease in cadence and an increase in right step length after the 3-month program. The step length in the right foot also showed a significant group × time interaction. Decreases in muscle mass, strength, power, and rate of force production are associated with a slower walking speed, shorter step length, and shorter swing phase during walking [21]. Previous randomized controlled trials using exercise interventions in older adults have reported increased step length and cadence [22, 23]. Indeed, our exercise intervention increased step length but decreased cadence in the intervention group. The subjects showed higher CWS and cadence at baseline compare to the older adults who participated a large cohort study [24]. The ceiling effect on CWS and cadence may have led decrease of those variables in accordance with increase of step length. Higher step length in the control group compared to the intervention group at baseline may also influence the results. Changes in gait are associated with age-related brain changes, including global brain atrophy, cerebral white matter lesions, and microbleeds [25, 26]. However, no studies have established a relationship between exercise intervention and brain activity, particularly glucose metabolism, during walking in older adults.

After the intervention, we observed a significant increase in regional brain glucose metabolism and gait performance during walking in older adults. In the intervention group, we observed a significant increase in regional glucose metabolism in the left posterior entorhinal cortex, left superior temporal gyrus, and right superior temporopolar area after the 3-month program. The entorhinal cortex is part of a critical pathway underlying memory formation. Zola-Morgan and colleagues reported that this area receives afferents from widespread association and limbic areas, including the hippocampus, and sends afferents back to the association neocortex and the dentate gyrus of the hippocampus [27]. Posterior portions of the entorhinal cortex receive projections carrying visuospatial information from the dorsal stream pathway via the parahippocampal cortex [28]. Visual feedback is required for locomotor adaptation [29] and is thought to override internal model predictions during locomotion [30]. Conversely, Zimmerman et al. found that increased variability in step length was associated with lower hippocampal metabolism in elderly individuals [31]. In their report, the authors suggested that the hippocampus could play an important role in the timing or rhythmicity of locomotion, which may be compromised in the elderly [31]. Unfortunately, in this study, we did not analyze the variance of walking parameters. Thus, further investigation regarding the relationship between walking performance and brain activity is warranted.

The association between impaired memory and medial temporal lobe atrophy, particularly in the hippocampus and entorhinal cortex, has been thoroughly established [32]. The pathological hallmarks of Alzheimer’s disease (e.g., neurofibrillary tangles and senile plaques) have been found in the entorhinal cortex in the earliest phase of the disease [33], and strategies to increase activity in that region may help prevent dementia in older adults.

We found that our exercise intervention was associated with increased glucose metabolism during walking in parts of the temporal lobe (the superior temporal gyrus and superior temporopolar area). A previous neuroimaging study indicated that disability in patients with Alzheimer’s disease could be associated with atrophy in temporal structures [34]. Our results suggest that activation of the temporal lobe during exercise may increase physical function and prevent future disability in older adults. A previous PET study found that the act of imagining walking along a path with obstacles was associated with increased prefrontal and parahippocampal activation. This suggests that higher brain centers are progressively and increasingly engaged when a locomotor task requires increased cognitive and sensory information processing [35]. To elucidate the role of exercise in activating specific brain areas, especially with the goal of increasing brain health and functional capacity among older adults, further experiments are warranted.

In the control group, we observed a relative increase in regional glucose metabolism in the left premotor and supplemental motor areas, left and right somatosensory association cortex, and right primary visual cortex after the 3-month period. The increased activity in supplemental motor area and prefrontal cortex reflect a compensation strategy in older adults [36]. Ensuring low gait variability, i.e. high gait performance, might require multisensory activation as old individuals with relative high gait variability show a significant relative deactivation of white matter of middle and superior temporal gyrus [37], pre-central gyrus [38] and supplemental motor area [37, 38] which is additionally accompanied with a lesser activity of the sensorimotor cortex [37]. Repeated measure might induce the compensatory activations in the peripheral region of supplemental motor area.

When comparing between the intervention and control groups, we found no significant increase in regional FDG uptake before vs. after the intervention. One of the reason which the intervention did not show between group differences might be short intervention period in the study. Thus, further research is needed to examine the relationship between such exercise interventions and regional glucose metabolism using long-term intervention design. Our study was limited in that we had a small sample size, which could have led to a type II error in the data analysis. A randomized controlled trial with a larger sample size may lead to a deeper understanding of the effects of exercise on physical function and brain activity. Further analysis adjusted potential confounding factors such as daily activity change is required to identify whether the intervention effects resulted from supervised program or activation of daily physical activity. Although older individuals with abnormal brain atrophy (e.g., severe atrophy) based on visual inspection by a radiologist were excluded, the brain atrophy levels could not be quantified. Age-related reduction of brain volume, especially in the entorhinal cortex and the temporal gyri, proceed [39, 40]. The effects of brain atrophy levels on the changes of brain activation between pre- and post-intervention should be considered. Furthermore, the loss of integrity in such normal-appearing white matter may play a role in causing gait disturbances [41]. Combined analysis of gray matter volume, integrity, and glucose metabolism may be useful to deep understand the effects of exercise on brain health [42].

## Conclusions

Our data revealed that exercise training does indeed alter regional brain activity, with relative increases observed in the precuneus and entorhinal cortices of older subjects. These regions play an important role in episodic and spatial memory formation. Further investigation to clarify the relationship between exercise and metabolic activity in the brain is warranted.

## Abbreviations

ANCOVA: Analysis of covariance
FDG: [^18^F]fluorodeoxyglucose
FEW: Family-wise error
fMRI: Functional magnetic resonance imaging
MNI: Montreal Neurological Institute
MRI: Magnetic resonance imaging
PET: Positron emission tomography
SPM: Statistical parametric mapping
